# Loss of Smi1, a protein involved in cell wall synthesis, extends replicative life span by enhancing rDNA stability in *Saccharomyces cerevisiae*

**DOI:** 10.1016/j.jbc.2021.100258

**Published:** 2021-01-07

**Authors:** Sujin Hong, Won-Ki Huh

**Affiliations:** 1 School of Biological Sciences, Seoul National University, Seoul, Republic of Korea; 2 Institute of Microbiology, Seoul National University, Seoul, Republic of Korea

**Keywords:** Smi1, Sir2, replicative life span (RLS), rDNA silencing, *Saccharomyces cerevisiae*, ChIP, chromatin immunoprecipitation, CLS, chronological life span, ERC, extrachromosomal ribosomal DNA circle, MAPK, mitogen-activated protein kinase, Pol, RNA polymerase, rDNA, ribosomal DNA, RENT, regulator of nucleolar silencing and telophase exit, RLS, replicative life span, SC, synthetic complete, STRE, stress response element, WT, wild-type

## Abstract

In *Saccharomyces cerevisiae*, replicative life span (RLS) is primarily affected by the stability of ribosomal DNA (rDNA). The stability of the highly repetitive rDNA array is maintained through transcriptional silencing by the NAD^+^-dependent histone deacetylase Sir2. Recently, the loss of Smi1, a protein of unknown molecular function that has been proposed to be involved in cell wall synthesis, has been demonstrated to extend RLS in *S. cerevisiae*, but the mechanism by which Smi1 regulates RLS has not been elucidated. In this study, we determined that the loss of Smi1 extends RLS in a Sir2-dependent manner. We observed that the *smi1*Δ mutation enhances transcriptional silencing at the rDNA locus and promotes rDNA stability. In the absence of Smi1, the stress-responsive transcription factor Msn2 translocates from the cytoplasm to the nucleus, and nuclear-accumulated Msn2 stimulates the expression of nicotinamidase Pnc1, which serves as an activator of Sir2. In addition, we observed that the MAP kinase Hog1 is activated in *smi1*Δ cells and that the activation of Hog1 induces the translocation of Msn2 into the nucleus. Taken together, our findings suggest that the loss of Smi1 leads to the nuclear accumulation of Msn2 and stimulates the expression of Pnc1, thereby enhancing Sir2-mediated rDNA stability and extending RLS in *S. cerevisiae*.

Two distinct life span paradigms have been proposed for *Saccharomyces cerevisiae* (1). Chronological life span (CLS), a model for the aging of postmitotic cells, is defined as the amount of time a cell can remain viable in a nondividing state (2). Replicative life span (RLS), a model for the aging of mitotic cells, is defined as the number of mitotic divisions that each mother cell can undergo before senescence (3). A primary cause of replicative aging in *S. cerevisiae* has been determined to be the accumulation of extrachromosomal ribosomal DNA circles (ERCs) (4). *S. cerevisiae* rDNA consists of 100–200 tandemly arrayed copies of a 9.1-kb repeat on chromosome XII (5, 6). Each rDNA repeat contains RNA polymerase (Pol) I-transcribed 35S rRNA and Pol III-transcribed 5S rRNA genes separated by the nontranscribed spacers NTS1 and NTS2. Since budding yeast has efficient homologous recombination systems, the highly repetitive rDNA array is an easy target for homologous recombination and is intrinsically unstable. Homologous recombination between rDNA repeats generates ERCs, which accumulate at toxic levels in mother cells, thereby limiting the RLS of mother cells (4). Therefore, ensuring the stability of rDNA is important for maintaining RLS in *S. cerevisiae*.

Under normal conditions, the stability of the rDNA array is maintained by transcriptional silencing. This mechanism, referred to as rDNA silencing, is mediated by the RENT (regulator of nucleolar silencing and telophase exit) complex, which suppresses Pol II-dependent transcription at the rDNA locus and inhibits homologous recombination between rDNA repeats (7). Sir2 is an NAD^+^-dependent histone deacetylase and a key subunit of the RENT complex (8, 9, 10). As a component of the RENT complex, Sir2 suppresses homologous recombination between rDNA repeats and the subsequent formation of ERCs, thereby extending RLS (4, 11, 12).

The association of Sir2 with rDNA and the enzymatic activity of Sir2 is regulated by endogenous levels of nicotinamide, a physiological inhibitor of Sir2 (13, 14). Nicotinamide is converted to nicotinic acid by the nicotinamidase Pnc1 as part of the NAD^+^ salvage pathway (15). Therefore, the deletion of *PNC1* increases intracellular nicotinamide levels and inhibits Sir2, while the overexpression of *PNC1* enhances Sir2 activity by reducing nicotinamide (16, 17, 18). The expression of Pnc1 is regulated by the stress-responsive transcription factors Msn2 and Msn4 *via* the stress response element (STRE), which is the Msn2/4 binding site in the *PNC1* promoter (15). Msn2/4 are normally maintained in the cytoplasm. However, various stress conditions, including glucose depletion, induce the nuclear accumulation of Msn2/4 (19, 20, 21). The translocation and nuclear accumulation of Msn2 are suppressed by the cAMP-PKA pathway and the TORC1 pathway and are activated by the protein kinases Mck1, Rim15, Yak1, Snf1, and Hog1, and the phosphatases Psr1, Psr2, and Glc7 (22). Hog1 is a mitogen-activated protein kinase (MAPK) that is involved in the high osmotic response pathway (23). Under hyperosmotic stress conditions, Hog1 is activated by phosphorylation and stimulates stress-responsive gene expression by phosphorylating both cytoplasmic and nuclear targets, including stress-responsive transcription factors (24, 25). Hog1 also responds to other stimuli, including heat shock, cold shock, ER stress, acetic acid, and arsenite (26, 27, 28, 29, 30).

Smi1 is a protein of unknown molecular function that has been hypothesized to be involved in the regulation of cell wall synthesis and the coordination of cell cycle progression with cell wall integrity (31, 32, 33). The loss of Smi1 leads to a 50% reduction in β-glucan synthase activity and β-glucan levels and hypersensitivity to cell wall damaging agents, such as caffeine, SDS, Congo red, Calcofluor white, and caspofungin (31, 34, 35, 36). Recently, an unexpected function of Smi1 in the regulation of the life span of *S. cerevisiae* has been reported. In a study of cell wall biosynthesis and longevity, it was observed that the loss of Smi1 significantly extends RLS (37). However, the mechanism by which Smi1 regulates RLS has not been elucidated.

In this study, we investigated the role of Smi1 in the regulation of RLS of *S. cerevisiae*. We observed that the loss of Smi1 extends RLS in a Sir2-dependent manner by enhancing transcriptional silencing at the rDNA locus and promoting rDNA stability. Smi1-deficient cells exhibited enhanced association of Sir2 with rDNA, elevated levels of Pnc1 protein, nuclear accumulation of Msn2, and increased binding of Msn2 to the *PNC1* promoter. We also observed that the activation of Pnc1 expression by Msn2 was regulated in a Hog1-dependent manner. Taken together, our results help to elucidate the molecular pathways involved in the regulation of RLS by Smi1 in *S. cerevisiae*.

## Results

### Loss of Smi1 extends RLS in a Sir2-dependent manner

**Figure 1:**
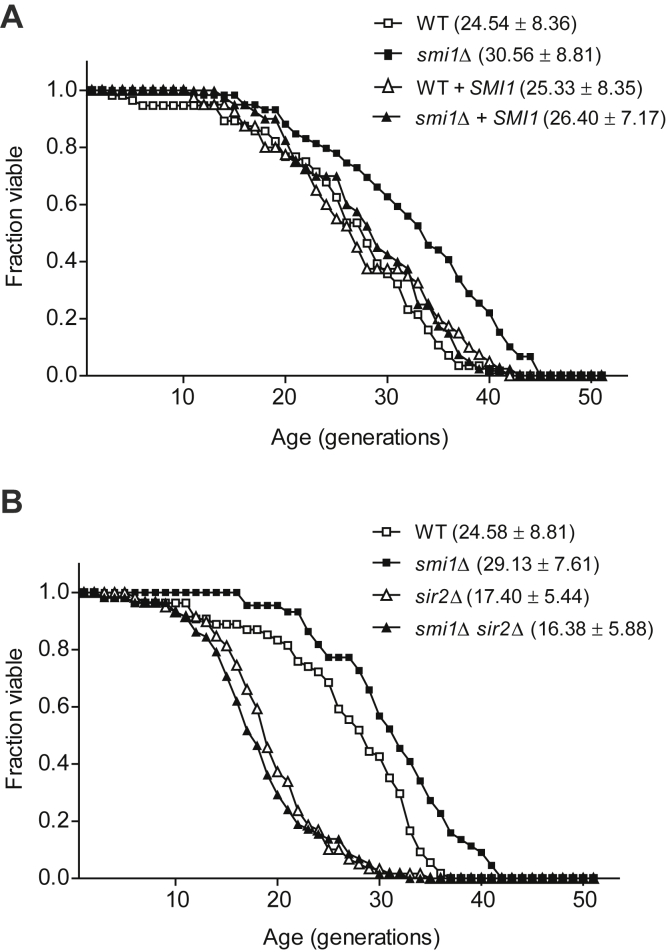
**Loss of Smi1 extends RLS in a Sir2-dependent manner.***A*, RLS analysis was performed with wild-type (WT), *smi1*Δ cells, and WT and *smi1*Δ cells containing chromosomally integrated *SMI1*. The *p*-values for *smi1*Δ cells, WT cells containing *SMI1* (WT + *SMI1*), and *smi1*Δ cells containing *SMI1* (*smi1*Δ + *SMI1*) *versus* WT cells are 7.1 × 10^−5^, 8.9 × 10^−1^, and 2.3 × 10^−1^, respectively. *B*, RLS analysis was performed with WT, *smi1*Δ, *sir2*Δ, and *smi1*Δ *sir2*Δ cells. The *p*-values for *smi1*Δ, *sir2*Δ, and *smi1*Δ *sir2*Δ cells *versus* WT cells are 1.2 × 10^−3^, 9.4 × 10^−7^, and 1.6 × 10^−7^, respectively. The *p*-value for *smi1*Δ *sir2*Δ cells *versus sir2*Δ cells is 2.1 × 10^−1^. Mean RLS values and standard deviations are shown in parentheses. RLS was determined from three independent experiments (approximately 60 cells per strain in total).

To investigate whether Smi1 regulates replicative aging in yeast, we measured the RLS of Smi1-deficient cells. The RLS of *smi1*Δ cells was extended by approximately 25% compared with that of wild-type cells (Fig. 1*A*). To confirm whether this alteration of RLS is truly *SMI1*-dependent, we introduced *SMI1* to Smi1-deficient cells and measured RLS. Re-expression of *SMI1* shortened the RLS of *smi1*Δ cells to a level similar to that of wild-type cells. Additional expression of *SMI1* in wild-type cells did not affect the RLS of wild-type cells. These results indicate that the loss of Smi1 extends RLS, even though the overexpression of Smi1 does not affect RLS.

Because Sir2 is a well-known factor for extending RLS (4), we subsequently investigated whether the RLS of *smi1*Δ cells was affected by Sir2. Consistent with the findings of a previous report (11), the RLS of Sir2-deficient cells was decreased by approximately 30% compared with that of wild-type cells (Fig. 1*B*). *smi1*Δ *sir2*Δ cells showed a similar RLS to that of *sir2*Δ cells. These results indicate that the loss of Smi1 increases RLS and that RLS extension in *smi1*Δ cells depends on the presence of Sir2.

### Loss of Smi1 increases Sir2-mediated rDNA silencing and promotes rDNA stability

**Figure 2:**
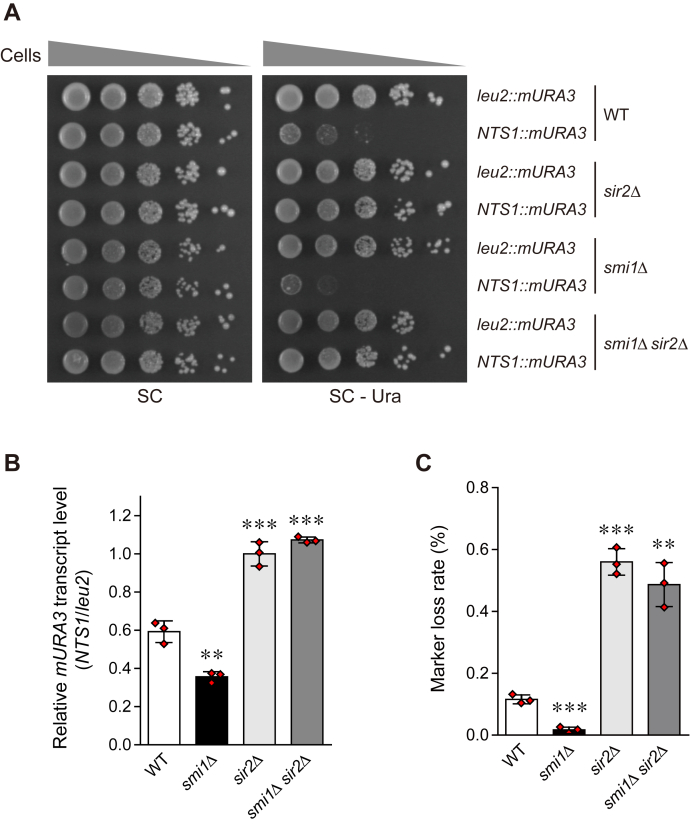
**Loss of Smi1 promotes rDNA stability in a Sir2-dependent manner.***A*, rDNA silencing assay was performed with wild-type (WT), *smi1*Δ, *sir2*Δ, and *smi1*Δ *sir2*Δ cells. Silencing at the rDNA region was assessed by monitoring the growth of tenfold serial dilution of cells on SC media lacking uracil. SC medium was used as a plating control. *B*, quantitative real-time reverse transcription-PCR analysis was performed to measure the *mURA3* transcript levels of the indicated cells. Amplification efficiencies were validated and normalized against *ACT1*. The relative transcript levels of the *mURA3* gene were calculated as the ratio of the normalized transcript levels of the *mURA3* gene inside the rDNA array (*NTS1::mURA3*) to that outside the rDNA array (*leu2::mURA3*). Values represent the average of three independent experiments, and error bars indicate the standard deviation. Asterisks indicate significant differences compared with WT cells (two-tailed Student's *t*-test): ∗∗∗*p* < 0.001, ∗∗*p* < 0.01. *C*, rDNA recombination assay was performed to check rDNA stability of the indicated cells. rDNA recombination is represented by the frequency of loss of the *ADE2* marker gene integrated at the rDNA locus in the corresponding cells. Values represent the average of three independent experiments, and error bars indicate the standard deviation. Asterisks indicate significant differences compared with WT cells (two-tailed Student's *t*-test): ∗∗∗*p* < 0.001, ∗∗*p* < 0.01.

Given that Sir2 increases rDNA stability by rDNA silencing and thus extends RLS (4, 11, 12), we surmised that RLS extension in Smi1-deficient cells may be attributable to increased rDNA stability. To examine whether the loss of Smi1 enhances rDNA stability, we conducted an rDNA silencing assay using yeast strains harboring the *mURA3* silencing reporter gene integrated either inside the NTS1 region of the rDNA locus (*RDN1-NTS1::mURA3*) or outside the rDNA locus (*leu2::mURA3*) (38). Cells were serially diluted tenfold and spotted onto SC medium as a plating control and SC medium without uracil to monitor *mURA3* silencing. In wild-type cells, the *mURA3* reporter gene was efficiently silenced at the rDNA locus (Figs. 2*A* and S1*A*). Compared with wild-type cells, *smi1*Δ cells showed enhanced transcriptional silencing of *mURA3* at the rDNA locus. In agreement with the RLS data, additional expression of *SMI1* in wild-type cells did not alter rDNA silencing, and re-expression of *SMI1* in *smi1*Δ cells restored the rDNA silencing of *smi1*Δ cells to a level similar to that of wild-type cells (Fig. S1*A*). As shown previously (38), transcriptional silencing at the rDNA locus was impaired in *sir2*Δ cells (Fig. 2*A*). *smi1*Δ *sir2*Δ cells exhibited impaired rDNA silencing similar to that of *sir2*Δ cells.

To confirm the spot assay results described above, we measured the transcription levels of the *mURA3* gene using real-time reverse transcription-PCR analysis. In wild-type cells, the transcription of the *mURA3* gene was effectively silenced at the rDNA locus compared with the outside of the rDNA locus (Fig. 2*B*). In Smi1-deficient cells, the transcription of the *mURA3* gene at the rDNA locus was further silenced. Consistent with the abovementioned spot assay results, transcriptional silencing of *mURA3* at the rDNA locus was abolished in *sir2*Δ and *smi1*Δ *sir2*Δ cells. These results suggest that the absence of Smi1 increases Sir2-mediated rDNA silencing.

We also examined rDNA stability by measuring the frequency of loss of the *ADE2* marker gene integrated at the rDNA locus (11). In keeping with the above observation that rDNA silencing was increased in *smi1*Δ cells, Smi1 deficiency decreased the rate of *ADE2* marker loss, and this phenotype was restored by re-expression of *SMI1* in *smi1*Δ cells (Figs. 2*C* and S1*B*). As reported previously (11), the absence of Sir2 significantly increased the rate of marker gene loss. *smi1*Δ *sir2*Δ cells exhibited an increased rDNA recombination rate that was similar to that of *sir2*Δ cells. This result indicates that the absence of Smi1 represses rDNA recombination and promotes rDNA stability. Taken together, these observations suggest that dysfunction of Smi1 increases yeast RLS by enhancing rDNA silencing and promoting rDNA stability in a Sir2-dependent manner.

### Loss of Smi1 enhances the association of Sir2 with the rDNA locus by increasing the expression of Pnc1 in an Msn2/4-dependent manner

**Figure 3:**
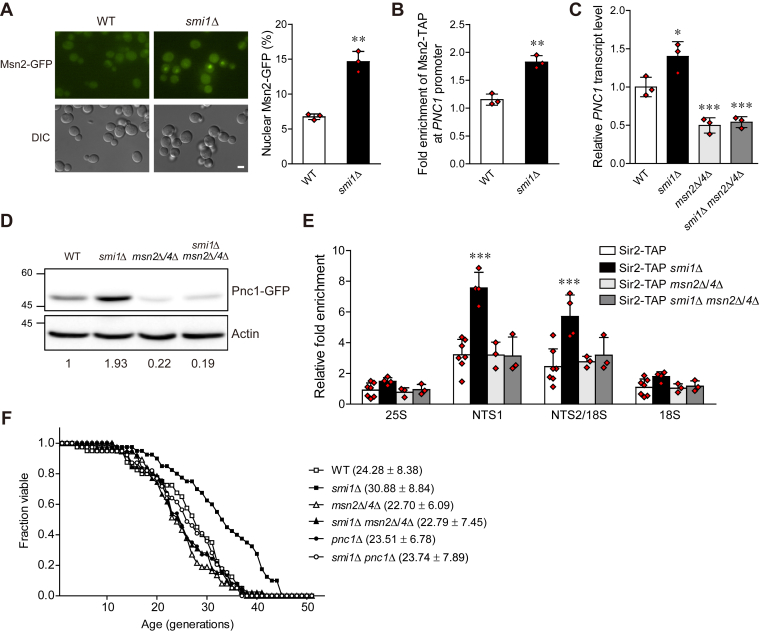
**Loss of Smi1 induces the nuclear accumulation of Msn2 and the expression of Pnc1, thereby enhancing the association of Sir2 with the rDNA region.***A*, cells with chromosomally GFP-tagged Msn2 were grown to logarithmic phase in SC medium and analyzed by fluorescence microscopy (left panel). Scale bars, 2 μm. The percentage of nuclear Msn2 in wild-type (WT) and *smi1*Δ cells was calculated (right panel). Values represent the average of three independent experiments, and at least 200 cells were counted for each determination. Error bars indicate the standard deviation. Asterisks indicate significant differences compared with the WT cells (two-tailed Student's *t*-test): ∗∗*p* < 0.01. *B*, the association of Msn2 with the *PNC1* promoter region was measured using a ChIP assay in WT and *smi1*Δ cells. Values represent the average of three independent experiments, and error bars indicate the standard deviation. Asterisks indicate significant differences compared with WT cells (two-tailed Student's *t*-test): ∗∗*p* < 0.01. *C*, quantitative real-time reverse transcription-PCR analysis was performed to measure the *PNC1* transcript levels of the indicated cells. Amplification efficiencies were validated and normalized against *ACT1*. The relative *PNC1* transcript levels were calculated as the ratio of the normalized transcript levels of *PNC1* to that of *ACT1*. Values represent the average of three independent experiments, and error bars indicate the standard deviation. *Asterisks* indicate significant differences compared with WT cells (two-tailed Student's *t*-test): ∗∗∗*p* < 0.001, ∗*p* < 0.05. *D*, immunoblotting was performed using a mouse anti-GFP antibody for the detection of GFP-tagged Pnc1 protein in the indicated cells. Actin was detected using an antiactin antibody and used as a loading control. The relative ratio of Pnc1 to actin was normalized against that of WT cells and is shown below each lane. The positions of molecular-weight markers (in kDa) are indicated on the left of blots. Data are representative of at least three independent experiments. *E*, the association of Sir2 with four representative regions in the rDNA locus (25S, NTS1, NTS2/18S, and 18S regions) was measured using a ChIP assay in the indicated cells. Values represent the average of at least three independent experiments, and error bars indicate the standard deviation. Asterisks indicate significant differences compared with WT cells (two-tailed Student's *t*-test): ∗∗∗*p* < 0.001. *F*, RLS analysis was performed with the indicated cells. The *p*-values for *smi1*Δ, *msn2*Δ/*4*Δ, *smi1*Δ *msn2*Δ/*4*Δ, *pnc1*Δ, and *smi1*Δ *pnc1*Δ cells *versus* WT cells are 1.5 × 10^−3^, 1.3 × 10^−1^, 2.0 × 10^−1^, 3.7 × 10^−1^, and 6.5 × 10^−1^, respectively. The *p*-value for *smi1*Δ *msn2*Δ/*4*Δ *versus msn2*Δ/*4*Δ cells is 9.7 × 10^−1^, and the *p*-value for *smi1*Δ *pnc1*Δ *versus pnc1*Δ cells is 5.5 × 10^−1^. Mean RLS values and standard deviations are shown in parentheses. RLS was determined from three independent experiments (approximately 60 cells per strain in total).

Msn2 and Msn4 are transcription factors that regulate the general stress response of *S. cerevisiae*. *MSN4* gene expression is induced by stress, while the expression of *MSN2* is constitutive (39). Under various stress conditions, nuclear-accumulated Msn2/4 binds to the STREs located in the promoters of stress-responsive genes and stimulates the expression of these genes (19). A previous study has shown that caloric restriction, which is known to prolong life span, enhances Sir2 activity and rDNA stability by inducing the translocation of Msn2/4 from the cytoplasm to the nucleus, where they increase the expression of nicotinamidase Pnc1, a Sir2 activator (18). Moreover, inhibition of TOR, another well-known condition that prolongs life span, also enhances rDNA stability by promoting the Msn2/4-Pnc1-Sir2 axis (14, 18). To investigate whether Msn2/4 is related to enhanced Sir2-mediated rDNA stability in Smi1-deficient cells, we examined the intracellular localization of Msn2/4. We constructed yeast strains in which the endogenous *MSN2* and *MSN4* genes were modified to produce a C-terminal GFP fusion protein and performed fluorescence microscopic analysis. In *smi1*Δ cells, Msn2 was significantly accumulated in the nucleus compared with wild-type cells, suggesting that the loss of Smi1 induces the translocation of Msn2 from the cytoplasm to the nucleus (Fig. 3*A*). We could not detect a GFP signal of Msn4 (data not shown), probably because of the low expression level of Msn4.

Given the above observations that the loss of Smi1 enhances rDNA stability and induces the nuclear accumulation of Msn2, we assumed that increased rDNA stability in Smi1-deficient cells might be mediated by Pnc1, which is upregulated by Msn2. To investigate whether nuclear-localized Msn2 binds to the *PNC1* promoter in Smi1-deficient cells, we constructed yeast strains in which the endogenous *MSN2* gene was modified to produce a C-terminal TAP fusion protein and conducted a chromatin immunoprecipitation (ChIP) assay. Notably, the loss of Smi1 increased the binding of Msn2 to the *PNC1* promoter (Fig. 3*B*). Next, we examined the expression levels of Pnc1 in Smi1-deficient cells. The transcript levels of *PNC1* were increased in *smi1*Δ cells (Fig. 3*C*). Consistent with this result, the protein levels of Pnc1 were also increased in *smi1*Δ cells (Fig. 3*D*). In the absence of Msn2/4, however, the loss of Smi1 did not increase the expression levels of Pnc1 (Fig. 3, *C* and *D*). These results suggest that Smi1 deficiency results in an increase in Pnc1 levels by stimulating its transcription and that the effect of Smi1 on *PNC1* expression is dependent on the presence of Msn2/4.

For the deacetylation reaction, Sir2 consumes NAD^+^ and produces *O*-acetyl-ADP ribose and nicotinamide. Nicotinamide is converted to nicotinic acid by the nicotinamidase Pnc1 as part of the NAD^+^ salvage pathway. Since nicotinamide is a noncompetitive inhibitor of Sir2, nicotinamide clearance by Pnc1 enhances the enzymatic activity of Sir2 (17). A previous study demonstrated that Pnc1 also enhances the association of Sir2 with rDNA and that the overexpression of *PNC1* suppresses rDNA recombination by promoting Sir2-mediated rDNA silencing (16). To examine whether elevated Pnc1 levels enhance the association of Sir2 with the rDNA locus in Smi1-deficient cells, we constructed yeast strains in which the endogenous *SIR2* gene was modified to produce a C-terminal TAP fusion protein and performed a ChIP assay for four representative regions of the rDNA locus—the 25S, NTS1, NTS2/18S, and 18S regions. Consistent with previous reports (7, 14), Sir2 was enriched at the NTS1 and NTS2/18S regions in wild-type cells (Fig. 3*E*). *smi1*Δ cells showed further enrichment of Sir2 at the NTS1 and NTS2/18S regions. The protein levels of Sir2 were not increased in *smi1*Δ cells (Fig. S2), indicating that the enrichment of Sir2 at the rDNA locus in *smi1*Δ cells is not due to increased Sir2 levels. Notably, the enhanced association of Sir2 with the rDNA locus in Smi1-deficient cells was abolished by the deletion of *MSN2/4*. Moreover, in keeping with the results described above, the loss of Smi1 did not extend the RLS in the absence of Pnc1 or Msn2/4 (Fig. 3*F*). Taken together, these observations suggest that Smi1 deficiency leads to enhanced association of Sir2 with the rDNA locus by increasing the expression of Pnc1 and extends RLS in an Msn2/4-dependent manner.

### Loss of Smi1 induces Msn2/4-dependent expression of Pnc1 by activating the MAP kinase Hog1

The localization of Msn2/4 is regulated by the cAMP-PKA signaling pathway. While upregulated PKA activity phosphorylates Msn2/4 and inhibits the import of Msn2/4 into the nucleus, downregulated PKA activity dephosphorylates Msn2/4 and mediates the nuclear accumulation of Msn2/4 (19, 22). To investigate whether the cAMP-PKA pathway regulates the nuclear localization of Msn2 in the absence of Smi1, we examined PKA activity in Smi1-deficient cells. For the determination of PKA activity, we employed a PKA substrate reporter derived from the native substrate Cki1 (40). PKA-dependent phosphorylation of the Cki1 reporter was detected by analyzing the mobility shift in SDS-PAGE. As reported previously (40, 41), in wild-type cells, we detected the phosphorylated forms of Cki1, which were significantly decreased under glucose starvation (Fig. S3*A*). *smi1*Δ cells did not exhibit a significant change in Cki1 phosphorylation compared with wild-type cells. This result suggests that the cAMP-PKA signaling pathway is not involved in the nuclear accumulation of Msn2 in Smi1-deficient cells.

It has been shown that the intracellular localization of Msn2/4 is also regulated by the TOR signaling pathway. Activated TORC1 induces cytoplasmic retention of Msn2/4 by stimulating the binding of Msn2/4 to the 14-3-3 protein Bmh2, while TORC1 inhibition induces the translocation of Msn2/4 from the cytoplasm to the nucleus (22, 42). To investigate whether the TOR signaling pathway controls the nuclear accumulation of Msn2 in the absence of Smi1, we examined TORC1 activity in *smi1*Δ cells. For the determination of TORC1 activity, the phosphorylation of Sch9, a major substrate of TORC1, was analyzed using an SDS-PAGE mobility shift assay (43). In keeping with the findings of previous reports (41, 43), the phosphorylated forms of Sch9 were observed in wild-type cells, and nitrogen starvation considerably reduced the phosphorylation of Sch9 (Fig. S3*B*). *smi1*Δ cells did not exhibit a significant change in Sch9 phosphorylation compared with wild-type cells. This result suggests that the TOR signaling pathway is not involved in the nuclear accumulation of Msn2 in the absence of Smi1.

**Figure 4:**
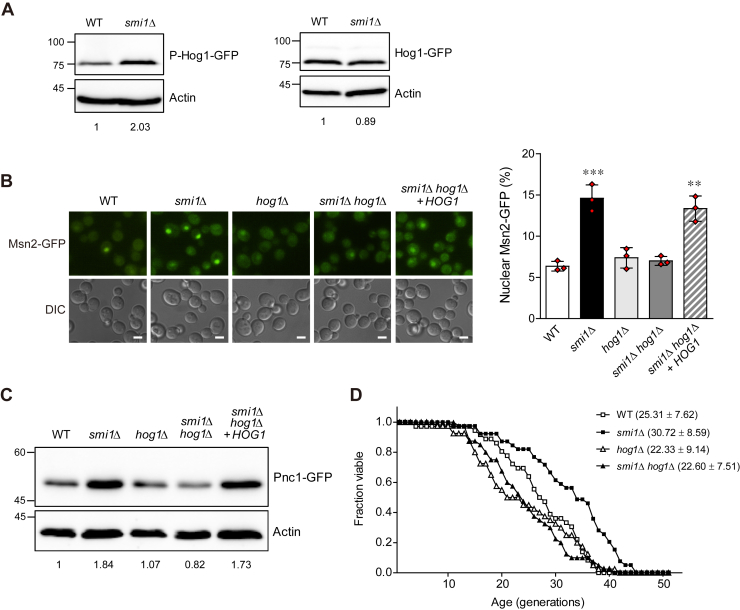
**Hog1 is required for the activation of Msn2 and the induction of Pnc1 expression in *smi1*Δ cells.***A*, total protein was extracted from WT and *smi1*Δ cells, and immunoblotting was performed using an anti-phospho-p38 MAPK (Thr180/Tyr182) antibody for the detection of phosphorylated Hog1 (left panel) and using an anti-GFP antibody for the detection of total Hog1 (right panel). Actin was detected using an antiactin antibody and used as a loading control. The relative ratio of phosphorylated Hog1 or total Hog1 to actin was normalized against that of WT cells and is shown below each lane. The positions of molecular-weight markers (in kDa) are indicated on the left of blots. Data are representative of at least three independent experiments. *B*, cells with chromosomally GFP-tagged Msn2 were grown to logarithmic phase in SC medium and analyzed by fluorescence microscopy (left panel). Scale bars, 2 μm. The percentage of nuclear Msn2 in WT, *smi1*Δ, *hog1*Δ, *smi1*Δ *hog1*Δ, and *smi1*Δ *hog1*Δ cells expressing *HOG1* in the pRS416 vector were calculated (right panel). Values represent the average of three independent experiments, and at least 200 cells were counted for each determination. Error bars indicate the standard deviation. *Asterisks* indicate significant differences compared with WT cells (two-tailed Student's *t*-test): ∗∗∗*p* < 0.001, ∗∗*p* < 0.01. *C*, total protein was extracted from WT, *smi1*Δ, *hog1*Δ, *smi1*Δ *hog1*Δ, and *smi1*Δ *hog1*Δ cells expressing *HOG1* in the pRS416 vector, and immunoblotting was performed using a mouse anti-GFP antibody for the detection of GFP-tagged Pnc1. Actin was detected using an antiactin antibody and used as a loading control. The relative ratio of Pnc1 to actin was normalized against that of WT cells and is shown below each lane. The positions of molecular-weight markers (in kDa) are indicated on the left of blots. Data are representative of at least three independent experiments. *D*, RLS analysis was performed with the indicated cells. The *p*-values for *smi1*Δ, *hog1*Δ, and *smi1*Δ *hog1*Δ cells *versus* WT cells are 3.0 × 10^−3^, 1.1 × 10^−1^, and 1.9 × 10^−1^, respectively. The *p*-value for *smi1*Δ *hog1*Δ *versus hog1*Δ cells is 4.9 × 10^−1^. Mean RLS values and standard deviations are shown in parentheses. RLS was determined from three independent experiments (approximately 60 cells per strain in total).

Msn2/4 activity is known to be modulated by several protein kinases, including Hog1 (22). Hog1 is a MAPK involved in the high osmotic response pathway (23). Under various stress conditions, Hog1 is activated by phosphorylation and stimulates stress-responsive gene expression by phosphorylating various cytoplasmic and nuclear targets including transcription factors Msn2/4 (24). To determine whether Hog1 is involved in the activation of Msn2 in the absence of Smi1, we examined the activation of Hog1 in Smi1-deficient cells by measuring the phosphorylation of Hog1. The loss of Smi1 increased the phosphorylation of Hog1 without altering the protein levels of Hog1 (Fig. 4*A*). These results suggest that the loss of Smi1 activates Hog1. Next, we investigated the role of Hog1 in the nuclear accumulation of Msn2. Notably, the nuclear accumulation of Msn2 induced by Smi1 deficiency was abolished in the absence of Hog1 (Fig. 4*B*), suggesting that the effect of Smi1 on the nuclear accumulation of Msn2 is dependent on the presence of Hog1. To determine whether the loss of Smi1 induces the nuclear localization of Msn2 in a Hog1-dependent manner, we introduced *HOG1* into *smi1*Δ *hog1*Δ cells and examined the nuclear accumulation of Msn2. Notably, re-expressing Hog1 in *smi1*Δ *hog1*Δ cells increased the nuclear accumulation of Msn2 to levels similar to those of *smi1*Δ cells (Figs. 4*B* and S4*A*)

Next, to investigate the role played by Hog1 in Msn2/4-mediated Pnc1 expression in *smi1*Δ cells, we examined the protein levels of Pnc1 in the absence of Hog1. The loss of Smi1 did not elevate Pnc1 expression in the absence of Hog1 (Fig. 4*C*), suggesting that the loss of Smi1 increases Pnc1 expression in a Hog1-dependent manner. In keeping with the nuclear accumulation of Msn2, re-expressing Hog1 in *smi1*Δ *hog1*Δ cells enhanced Pnc1 expression to levels similar to those of Smi1-deficient cells (Figs. 4*C* and S4B). In addition, in keeping with the data described above, the loss of Smi1 did not extend the RLS in the absence of Hog1 (Fig. 4*D*). Taken together, our observations suggest that the loss of Smi1 stimulates the expression of Pnc1 by translocating Msn2 from the cytoplasm to the nucleus, thereby extending RLS, and that this regulation is mediated by Hog1, not by the cAMP-PKA or the TOR signaling pathway.

## Discussion

Smi1 is known to be a transcriptional modulator of gene expression involved in cell wall biosynthesis and maintenance (31, 35), but its function has not been elucidated in detail. Smi1 has 533 genetic and 42 physical interactions with different partners, most of which are involved in the process of cell wall biosynthesis and maintenance (32, 33, 44, 45, 46, 47). Given that other interaction partners of Smi1 are involved in various cellular processes, such as the cell cycle, metabolism, and osmoregulation (44, 46, 47), it is likely that Smi1 plays a role in cellular processes other than maintaining cell wall integrity. In this study, we demonstrated that Smi1 regulates the cellular aging process in *S. cerevisiae*; the loss of Smi1 extends RLS by promoting Sir2-mediated rDNA silencing and rDNA stability. It is notable for a protein involved in the regulation of cell wall synthesis and maintenance to play a role in rDNA silencing and RLS regulation. Similar cases have been reported previously. For instance, the loss of Gas1, a β-1,3-glucanosyltransferase that participates in the formation and maintenance of β-1,3-glucan chains and cell wall assembly, increases Sir2-mediated rDNA silencing (48, 49). It has also been reported that treatment with Congo red, a cell-wall-damaging agent, increases rDNA silencing in a Sir2-dependent manner (49). These studies provide a link between cell wall stress and transcriptional silencing at the rDNA region and suggest the hypothesis that the loss of Smi1 causes cell wall stress that leads to RLS extension by enhancing rDNA silencing. On the other hand, we also obtained results indicating that the role of Smi1 in rDNA silencing and RLS regulation is not related to cell wall integrity. First, treatment with 1 M sorbitol, which is known to relieve cell wall stress (50, 51), did not affect rDNA silencing in *smi1*Δ cells (Fig. S5*A*). Second, while a defect in the β-1,3-glucan structure is a common feature of the *gas1*Δ mutant, *smi1*Δ mutant, and cells under Congo red treatment (35, 52, 53, 54), the loss of Fks1, a catalytic subunit of β-1,3-glucan synthase, was not observed to affect rDNA silencing (Fig. S5*B*). In addition, several cell-wall-perturbing agents, such as Calcofluor white, caffeine, SDS, and vanadate, have not been observed to affect transcriptional silencing at the rDNA region (49). Taken together, these findings suggest that enhanced rDNA silencing in the *smi1*Δ mutant is achieved through the loss of Smi1 function, which is irrelevant to cell wall biosynthesis and maintenance. It is possible that Gas1 also shares this feature of Smi1 in the regulation of rDNA silencing.

In the absence of Smi1, Msn2 translocates from the cytoplasm to the nucleus. Nuclear-accumulated Msn2 stimulates the expression of Pnc1, an activator of Sir2, thereby enhancing rDNA silencing. This regulatory mechanism, known as the Msn2/4-Pnc1-Sir2 axis, has been reported previously (14, 18). Unfortunately, we were not able to detect the localization of Msn4, probably because the endogenous expression levels of Msn4 were too low to be detected by fluorescence microscopy. Msn2 and Msn4 are partially redundant, and the contributions of Msn2 and Msn4 to Pnc1 expression may be different. To examine the contributions of Msn2 and Msn4 to Pnc1 expression, we determined the protein levels of Pnc1 in *smi1*Δ *msn2*Δ and *smi1*Δ *msn4*Δ cells. The absence of Msn2 significantly reduced Pnc1 expression, but the absence of Msn4 did not affect Pnc1 expression in *smi1*Δ cells (Fig. S6). In keeping with this observation, a previous study showed that while the deletion of *MSN2* impairs caloric restriction-induced expression of *PNC1*, the deletion of *MSN4* has little effect (18). All these results suggest that Msn2 plays a major role in Pnc1 expression in the absence of Smi1.

Under zymolyase treatment, cell wall stress induces transcription of various genes. A previous study demonstrated that two MAP kinases, Slt2 and Hog1, cooperate in this transcriptional regulation process (55). In this process, the transcription of genes involved in various functions, such as metabolism, is regulated in addition to the expression of genes related to cell wall synthesis and maintenance. Among these genes that are transcriptionally regulated in a Hog1-dependent manner are several Msn2 target genes (55, 56). In this study, we found that Hog1 is activated in *smi1*Δ cells and that the activation of Hog1 results in the translocation of Msn2 into the nucleus, but how the loss of Smi1 activates Hog1 has not been determined. Further research is warranted to elucidate the regulatory mechanisms governing Hog1 activation in *smi1*Δ cells.

The function of Hog1 has been well studied under osmotic stress conditions. Under high external osmolarity, Hog1 is phosphorylated and translocates from the cytoplasm to the nucleus to promote stress-responsive gene expression either directly or by phosphorylating other transcription factors (24, 25, 57). Under other stress conditions, however, it has also been reported that Hog1-dependent stress response can occur without the nuclear localization of Hog1 (28, 58, 59). We observed that, while Smi1 deficiency led to a significant increase in the phosphorylation of Hog1 (Fig. 4*A*), the portion of *smi1*Δ cells with nuclear-localized Hog1 was very small (data not shown). A similar phenomenon has been reported under prolonged ER stress conditions. Bicknell *et al.* observed that, under prolonged ER stress conditions, Hog1 phosphorylation was sustained even though Hog1 was retained in the cytosol (59). Given that only a small portion of *smi1*Δ cells exhibits nuclear-localized Hog1, it seems that Hog1 activation in *smi1*Δ cells is not related to its nuclear accumulation. Further studies are needed to elucidate how cytosolic Hog1 activated by Smi1 deficiency regulates the activity of Msn2/4.

It is widely accepted that genes belonging to similar biological processes share common genetic interactions, and genes encoding proteins that function within the same pathway or complex display similar genetic interaction profiles (60). Several genes exhibit genetic interaction profiles similar to those of *SMI1* (61). While some of these genes—*FKS1*, *GAS1*, *KRE1*, *CCW1*, *RHO1*, *ACT1*, and *SLA1*—have functions related to cell wall biosynthesis and morphology, the functions of other genes—*PBR1*, *AIM26*, *AIM44*, *ILM1*, and *PAL1*—have not been elucidated. Given that genes with similar genetic interaction profiles are expected to have related functions, it will be interesting to further investigate whether the abovementioned genes with interaction profiles similar to those of *SMI1* can also regulate rDNA silencing and RLS. This approach may help to elucidate the regulatory mechanisms governing cellular aging in yeast.

Although Smi1 does not have a mammalian homolog, Hog1, Msn2/4, and Sir2 are conserved from yeast to human, and Pnc1 also has a functional ortholog in human. In particular, SIRT7, a mammalian homolog of Sir2, protects against mammalian cellular senescence induced by rDNA instability (62). SIRT1, another mammalian homolog of Sir2, is also known to play an antiaging role, although there is no report yet demonstrating that it affects rDNA stability like Sir2 in yeast (63). In addition, NAMPT1, a functional ortholog of Pnc1, helps SIRT1's antiaging functions in the same way as Pnc1 in yeast (63, 64). In mammals, p38 and JNK, which are Hog1 homologs, are involved in complex signaling pathways that have both pro- and antiaging roles (65, 66). rDNA instability is also observed in mammals and is involved in various diseases such as cancer as well as aging (67, 68, 69). Therefore, studies on the mechanisms regulating rDNA stability in yeast, like this study, will provide new insights into understanding aging and diseases in mammals.

## Experimental procedures

### Yeast strains and growth media

The yeast strains utilized in this study are listed in Table S1. Yeast cells were grown in YPD medium (1% yeast extract, 2% peptone, and 2% glucose) or synthetic complete (SC) medium (0.67% yeast nitrogen base without amino acids, 2% glucose, and nutritional supplements) lacking appropriate amino acids for selection. All cultures were incubated at 30 °C. Gene disruption was carried out using the one-step PCR-based gene targeting procedure, as previously described (70). Yeast transformation was performed using the lithium acetate method (71), and proper integration was confirmed by PCR. For the expression of Smi1, DNA fragments containing *SMI1-Myc* sequences were amplified by PCR and cloned into the pRS303 vector with its own promoter, digested with *Pst*I and integrated into the *HIS3* locus. For the construction of the Hog1-expressing plasmids, DNA fragments containing *HOG1-Myc* sequences were amplified by PCR and cloned into the pRS416 vector with the *ADH1* promoter.

### RLS analysis

Analysis of RLS was conducted by micromanipulation as described previously (72) using a Zeiss Tetrad microscope. All measurements of RLS were performed on YPD plates. RLS was determined from three independent experiments (approximately 60 cells per strain in total). For statistical analysis, RLS data sets were compared by a Wilcoxon rank-sum test. Cells that never budded were excluded from the analysis.

### rDNA silencing assay

Silencing at the rDNA region was tested as described previously (38, 73). Yeast cells were grown to an OD_600_ of 0.8, and 2.5 μl of tenfold serial dilutions of the cell suspensions was spotted onto the appropriate media. Plates were incubated at 30 °C for 2 days before visualization.

### Quantification of mURA3 and PNC1 transcript level

Total RNA was extracted from yeast cells using the RNeasy Mini Kit (Qiagen). cDNA for reverse transcription-PCR was generated using the ReverTra Ace qPCR RT Kit (TOYOBO). The amounts of *mURA3*, *PNC1*, and *ACT1* transcripts were analyzed by quantitative real-time reverse transcription-PCR using the QuantStudio 3 Real-Time PCR System (Applied Biosystems) and the 2X Real-Time PCR Kit with SFCgreen I (BioFACT). The primers used for the amplification of *mURA3*, *PNC1*, and *ACT1* are shown in Table S2. The silencing reporter gene *mURA3*, which contains the *TRP1* promoter instead of the *URA3* promoter, has been described previously (74). For the calculation of the relative *mURA3* transcript level, the transcript levels of the *mURA3* reporter gene inside or outside the rDNA locus were measured and normalized against that of *ACT1*. The ratio of normalized *mURA3* transcript levels inside (*NTS1::mURA3*) to those outside (*leu2::mURA3*) was calculated. The transcript level of *PNC1* was measured and normalized against that of *ACT1* and quantified by the 2^−ΔΔC^_T_ method (75). All relative transcript levels were determined from three independent experiments and analyzed statistically by a two-tailed Student's *t*-test.

### rDNA recombination assay

The rDNA recombination rate was determined by measuring the frequency of the loss of *ADE2* integrated at the rDNA locus of strain DMY3010 as described previously (11). Yeast cells were grown to an OD_600_ of ∼1.0 in SC medium and spread on SC plates. Colonies were grown for 2 days at 30 °C and then placed at 4 °C for 2 days to improve color development. The rDNA recombination rate was calculated by dividing the number of half-red/half-white colonies by the number of total colonies. For each assay, more than 10,000 colonies were examined. Entirely red colonies were excluded from all calculations. Three independent experiments were performed and analyzed statistically by a two-tailed Student's *t*-test.

### Fluorescence microscopy

Fluorescence microscopy was performed on a Nikon Eclipse E1 microscope with a Plan Fluor 100×/1.30 NA oil immersion objective. Image analysis was performed using NIS Elements imaging software (Nikon). The percentage of cells with predominately nuclear fluorescence was determined from three independent experiments. Approximately 200 cells were counted for each experiment and were analyzed statistically by a two-tailed Student's *t*-test.

### ChIP assay

ChIP assays were performed as previously described (7). Yeast cultures (100 ml) were grown to an OD_600_ of 1.2∼1.5 and cross-linked with 1% formaldehyde for 15 min. The reaction was quenched with glycine at a final concentration of 125 mM for 5 min. Cells were washed twice with cold phosphate-buffered saline. Cell pellets were resuspended in 400 μl of lysis buffer (50 mM HEPES-KOH at pH 7.5, 500 mM NaCl, 1 mM EDTA at pH 8.0, 1% Triton X-100, 0.1% sodium deoxycholate, 0.1% SDS, 1 mM phenylmethylsulfonyl fluoride, 1 mM benzamidine, 1 mg/ml leupeptin, and 1 mg/ml pepstatin) and bead-beaten with 0.5-mm glass beads ten times for 1 min at 4 °C. Samples were incubated on ice for 2 min between bead beatings. Cell lysates were sonicated ten times for 15 s with amplitude set at 15% and centrifuged twice at 13,000 rpm for 10 min at 4 °C. The DNA concentration of each cell lysate was quantified and diluted to 2 mg/ml with lysis buffer. To obtain input DNA, 100 μl of lysate was used for each immunoprecipitation reaction. For ChIP experiments, 100 μl of a 50% slurry of prewashed IgG-agarose beads (Amersham Biosciences) was incubated with 1 ml of lysate at 4 °C overnight. Beads were washed twice in lysis buffer, once with wash buffer (10 mM Tris-HCl at pH 8.0, 0.25 M LiCl, 0.5% NP-40, 0.5% sodium deoxycholate, and 1 mM EDTA at pH 8.0), and once with TE buffer at room temperature. Beads were eluted by adding 50 μl of elution buffer (50 mM Tris–HCl at pH 8.0, 10 mM EDTA, and 1% SDS) and incubating at 65 °C for 10 min. Eluate was transferred to a fresh tube and pooled with a final bead wash of 150 μl of elution buffer. For input DNA, 100 μl of elution buffer was added to 100 μl of lysate. Input and ChIP samples were incubated at 65 °C overnight, combined with 300 μl of TE and 250 μg of proteinase K, and incubated at 37 °C for 2 h. All samples were extracted once with phenol:chloroform:isoamyl alcohol and once with chloroform. NaOAc (at a final concentration of 300 mM), and 20 μg of glycogen was added to 400 μl of sample. Precipitated and washed DNA was resuspended in 100 μl of TE with 1 μg of RNase A and incubated at 37 °C for 1 h. ChIP samples were analyzed by quantitative real-time PCR using the QuantStudio 3 Real-Time PCR System (Applied Biosystems) and the 2X Real-Time PCR Kit with SFCgreen I (BioFACT). The primers used for PCR are shown in Table S3. Three independent experiments were performed and analyzed statistically by a two-tailed Student's *t*-test.

### Western blot analysis

Exponentially growing cells were lysed by suspending cells in lysis buffer with protease inhibitors (50 mM Tris-HCl at pH 7.5, 150 mM NaCl, 0.01% NP-40, 1 mM EDTA, 1 mM phenylmethylsulfonyl fluoride, 1 mM benzamidine, 1 μg/ml leupeptin, and 1 μg/ml pepstatin), followed by bead beating. Lysates were clarified by centrifugation at 13,000 rpm for 10 min at 4 °C. Protein concentration was determined by Bradford assay. Protein samples were mixed with 6× SDS sample buffer and boiled for 3 min at 95 °C. SDS-PAGE was performed with 8% separating gel. Western blot analysis was performed by standard methods using an HRP-conjugated mouse anti-GFP antibody (sc-9996, Santa Cruz Biotechnology) and an HRP-conjugated antimouse IgG antibody (A9044, Sigma). Actin was used as a loading control and was detected by HRP-conjugated mouse antiactin antibody (sc-40, Santa Cruz Biotechnology). Images were captured using a luminescent image analyzer, AE-9150 Ez-Capture II (ATTO), and quantified using CS analyzer version 3.0 software (ATTO).

### Measurement of protein phosphorylation

Analysis of phosphorylated Cki1 and phosphorylated Hog1 was conducted by western blotting, as described previously (40). Cells were grown to an OD_600_ of ∼1.0 and lysed by suspending cells in lysis buffer with protease inhibitors and phosphatase inhibitors (50 mM Tris-HCl, pH 7.5, 150 mM NaCl, 0.01% NP-40, 1 mM EDTA, 1 mM phenylmethylsulfonyl fluoride, 1 mM benzamidine, 1 μg/ml leupeptin, 1 μg/ml pepstatin, 10 mM sodium fluoride, 10 mM β-glycerolphosphate, 10 mM sodium orthovanadate, and 10 mM sodium pyrophosphate) followed by bead beating. Lysates were clarified by centrifugation at 13,000 rpm for 10 min at 4 °C. Protein concentration was determined by Bradford assay. Protein samples were mixed with 6× SDS sample buffer and boiled for 3 min at 95 °C. SDS-PAGE was performed with 8% separating gel. Cki1 phosphorylation was detected by SDS-PAGE and immunoblotting using an HRP-conjugated mouse anti-Myc antibody (sc-40, Santa Cruz Biotechnology). Hog1 phosphorylation was detected by SDS-PAGE and immunoblotting using an anti-phospho-p38 MAPK (Thr180/Tyr182) antibody (#4511, Cell Signaling Technology). Actin was used as a loading control and was detected by an HRP-conjugated mouse antiactin antibody (sc-40, Santa Cruz Biotechnology). Analysis of phosphorylated Sch9 was conducted by western blotting, as described previously (43). Cells were grown to an OD_600_ of ∼1.0, and trichloroacetic acid was added up to 6%. Samples were placed on ice for 10 min, spun down, washed twice with cold acetone, and dried. Cells were bead-beaten in 100 μl of urea buffer with protease inhibitors and phosphatase inhibitors (6 M urea, 50 mM Tris-HCl, pH 7.5, 5 mM EDTA, 1% SDS, 0.1 mM phenylmethylsulfonyl fluoride, 10 mM sodium fluoride, 10 mM β-glycerolphosphate, 10 mM sodium orthovanadate, and 10 mM sodium pyrophosphate) followed by heating for 10 min to 65 °C. For 2-nitro-5-thiocyanobenzoic acid cleavage, 30 μl of 0.5 M cyclohexylamino ethane sulfonic acid, pH 10.5, and 20 μl of 7.5 mM 2-nitro-5-thiocyanobenzoic acid were added, and samples were incubated overnight at room temperature. Protein samples were mixed with 6× SDS sample buffer, boiled for 10 min at 65 °C, and clarified by centrifugation at 13,000 rpm for 1 min. SDS-PAGE was performed with an 8% separating gel. Sch9 phosphorylation was detected by SDS-PAGE and immunoblotting using an HRP-conjugated mouse anti-HA antibody (sc-7392 HRP, Santa Cruz Biotechnology). Actin was used as a loading control and was detected by HRP-conjugated mouse antiactin antibody (sc-40, Santa Cruz Biotechnology).

## Data availability

All data are contained within the article.

## Conflict of interest

The authors declare that they have no conflicts of interest with the contents of this article.
