# Supplementary material for: Loss of Smi1, a protein involved in cell wall synthesis, extends replicative life span by enhancing rDNA stability in Saccharomyces cerevisiae
Source: J Biol Chem. 2021 Jan 7;296:100258. doi: 10.1016/j.jbc.2021.100258 (PMC7948926; doi:10.1016/j.jbc.2021.100258)
Supplement: Supporting information [file mmc1.pdf]

Supporting Information

Loss of Smi1, a protein involved in cell wall synthesis, extends replicative lifespan by enhancing rDNA stability in *Saccharomyces cerevisiae*

Sujin Hong[1] and Won-Ki Huh[1,2]*

[1]School of Biological Sciences, Seoul National University, Seoul 08826, Republic of Korea.

[2]Institute of Microbiology, Seoul National University, Seoul 08826, Republic of Korea

* For correspondence: Won-Ki Huh, wkh@snu.ac.kr.

Table of Contents

Figure S1. Loss of Smi1 enhances rDNA silencing and rDNA stability

Figure S2. Loss of Smi1 does not significantly alter the protein levels of Sir2.

Figure S3. cAMP-PKA signaling pathway or TOR signaling pathway is not involved in the activation of Msn2 in *smi1*Δ cells.

Figure S4. Loss of Smi1 induces the activation of Msn2 and the expression of Pnc1 in a Hog1-dependent manner.

Figure S5. The role of Smi1 in rDNA silencing is not related to cell wall integrity.

Figure S6. Msn4 plays a minor role in Pnc1 expression.

Table S1. Strains used in this study.

Table S2. Oligonucleotide primers used for quantitative real-time PCR in this study.

Table S3. Oligonucleotide primers used for ChIP assays in this study.

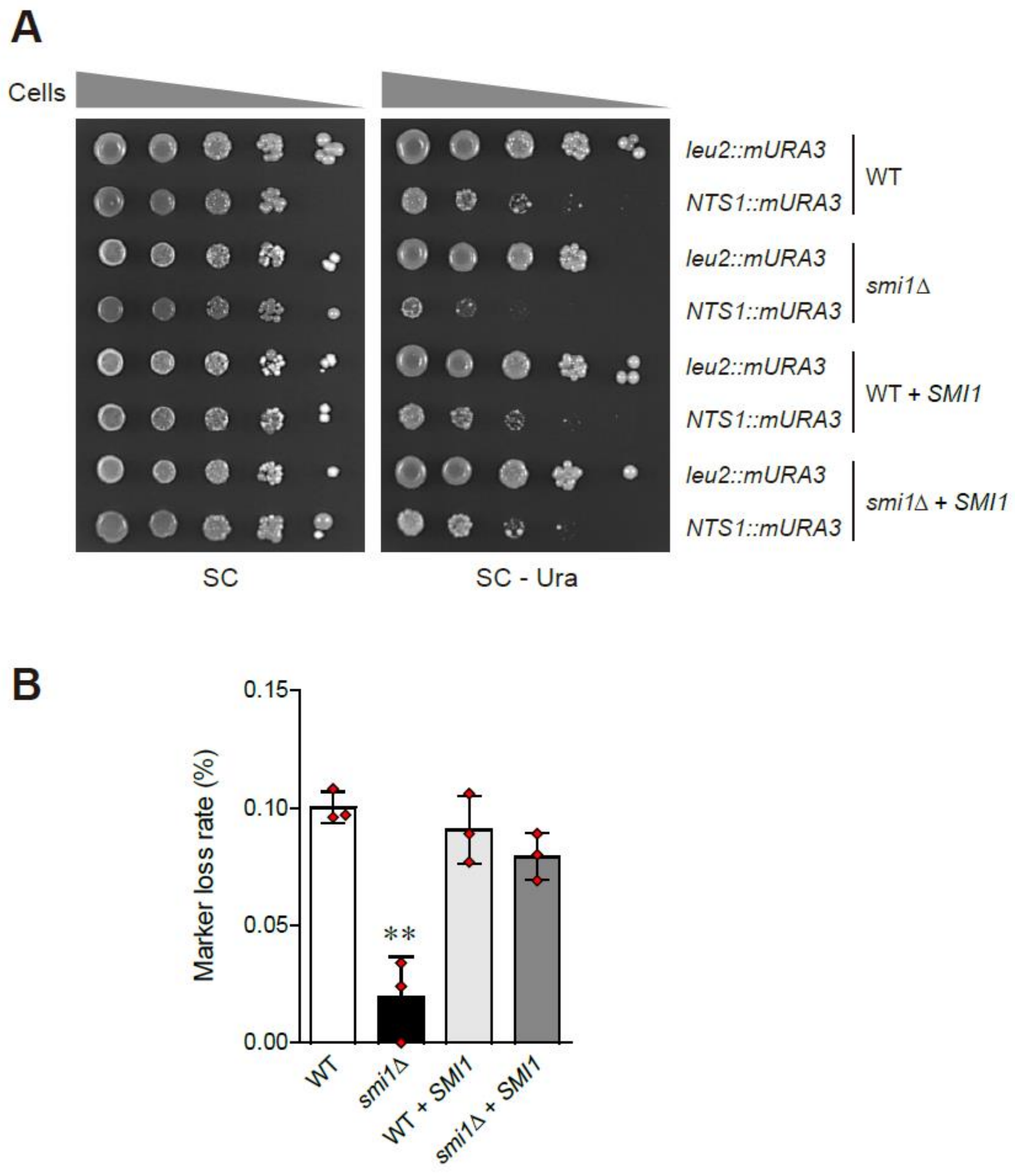

Figure S1. Loss of Smi1 enhances rDNA silencing and rDNA stability. *A,* rDNA silencing assay was performed with wild-type (WT), *smi1*Δ cells, and WT and *smi1*Δ cells containing chromosomally integrated *SMI1*. Silencing at the rDNA region was assessed by monitoring the growth of tenfold serial dilution of cells on SC media lacking uracil. SC medium was used as a plating control. *B,* rDNA recombination assay was performed to check rDNA stability of the indicated cells. rDNA recombination is represented by the frequency of loss of the *ADE2* marker gene integrated at the rDNA locus in the corresponding cells. Values represent the average of three independent experiments, and error bars indicate the standard deviation. Asterisks indicate significant differences compared with WT cells (two-tailed Student's *t*-test) $^{**}P < 0.01$.

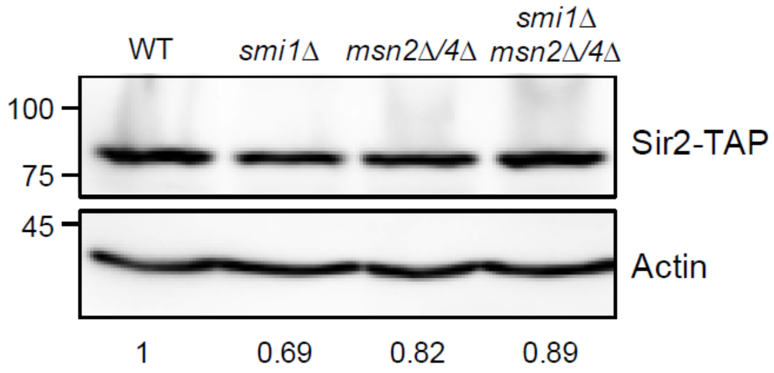

Figure S2. Loss of Smi1 does not significantly alter the protein levels of Sir2. Total protein was extracted from wild-type (WT), *smi1*Δ, *msn2*Δ/*4*Δ, and *smi1*Δ *msn2*Δ/*4*Δ cells, and immunoblotting was performed using an anti-mouse IgG antibody for the detection of TAP-tagged Sir2 protein. Actin was detected using an anti-actin antibody and used as a loading control. The relative ratio of Sir2 to actin was normalized against that of WT cells and is shown below each lane. The positions of molecular-weight markers (in kDa) are indicated on the left of blots. Data are representative of at least three independent experiments.

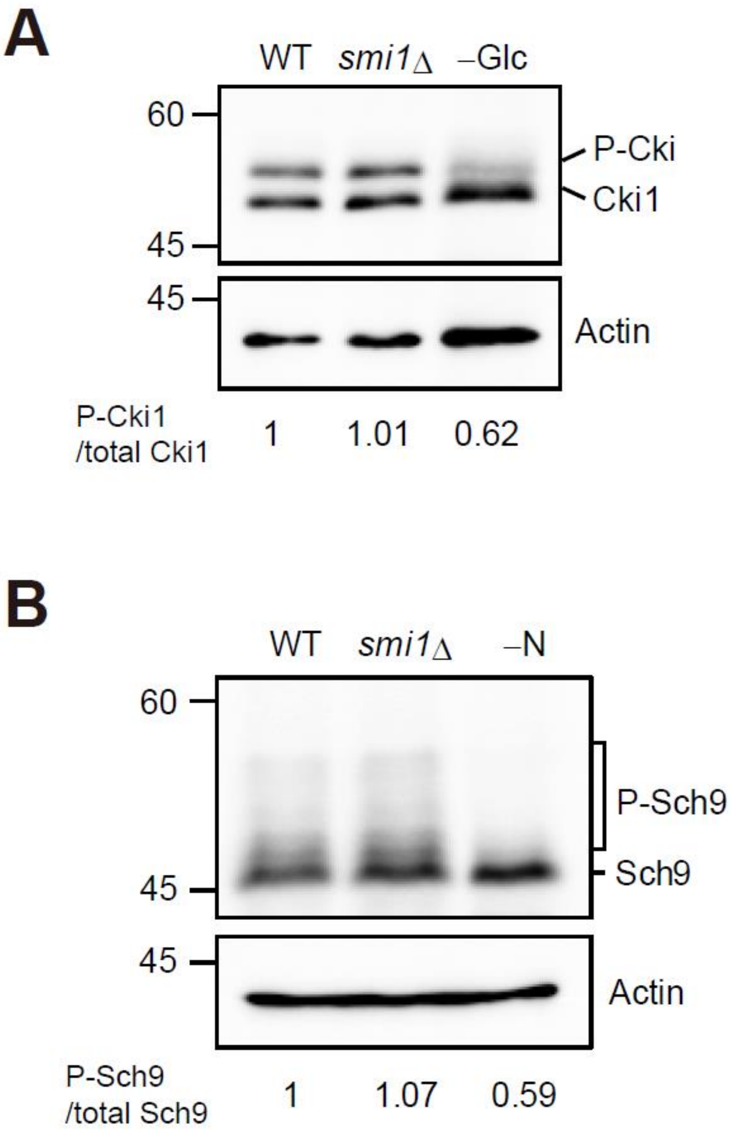

Figure S3. cAMP-PKA signaling pathway or TOR signaling pathway is not involved in the activation of Msn2 in *smi1*Δ cells. *A,* Total protein was extracted from wild-type (WT), *smi1*Δ, and WT cells under glucose starvation. All cells harbor *pRS423-*P_{CUP1}*-6xMYC-cki1*$^{2\text{-}200(S125/130A)}$. Immunoblotting was performed using a mouse anti-Myc antibody for the detection of Myc-tagged Cki1 protein. Actin was detected using an anti-actin antibody and used as a loading control. The relative ratio of phosphorylated Cki1 to total Cki1 is shown below each lane. The positions of molecular-weight markers (in kDa) are indicated on the left of blots. Data are representative of at least three independent experiments. *B,* Total protein was extracted from WT, *smi1*Δ, and WT cells under nitrogen starvation. All cells harbor *pRS416-SCH9*^{T570A}*-5HA*. Immunoblotting was performed using a mouse anti-HA antibody for the detection of HA-tagged Sch9 protein. Actin was detected using an anti-actin antibody and used as a loading control. The relative ratio of phosphorylated Sch9 to total Sch9 is shown below each lane. The positions of molecular-weight markers (in kDa) are indicated on the left of blots. Data are representative of at least three independent experiments.

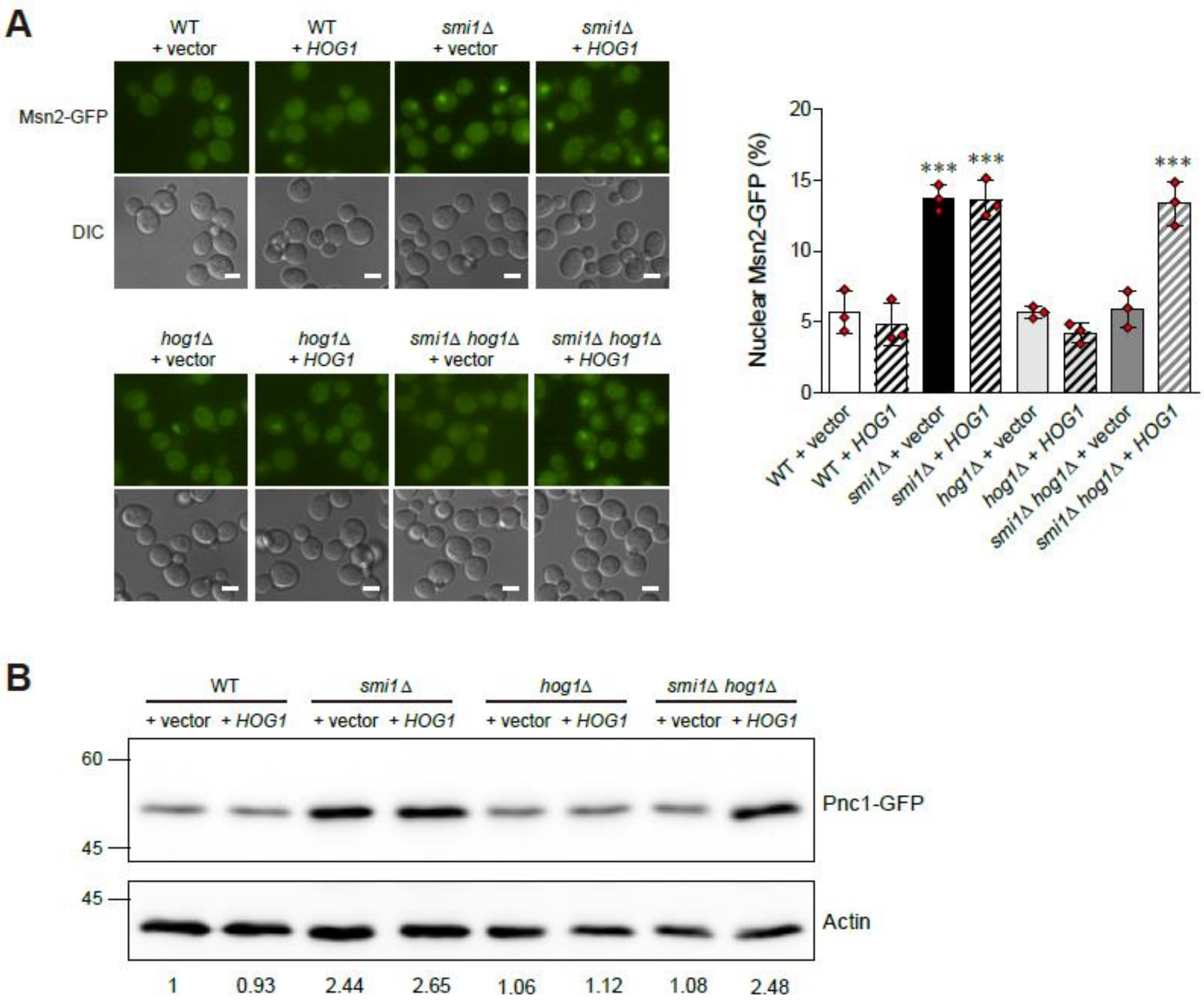

Figure S4. Loss of Smi1 induces activation of Msn2 and the expression of Pnc1 in a Hog1-dependent manner. *A,* Cells with chromosomally GFP-tagged Msn2 were grown to logarithmic phase in SC medium and analyzed by fluorescence microscopy (left panel). Scale bars, 2 μm. The percentage of nuclear Msn2 in wild-type (WT), *smi1*Δ, *hog1*Δ, and *smi1*Δ *hog1*Δ cells expressing *HOG1* in the pRS416 vector or carrying empty vector were calculated (right panel). Values represent the average of three independent experiments and at least 200 cells were counted for each determination. Error bars indicate the standard deviation. Asterisks indicate significant differences compared with WT cells (two-tailed Student's *t*-test): ***$P < 0.001$. *B,* Total protein was extracted from WT, *smi1*Δ, *hog1*Δ, and *smi1*Δ *hog1*Δ cells expressing *HOG1* in the pRS416 vector or carrying empty vector, and immunoblotting was performed using a mouse anti-GFP antibody for the detection of GFP-tagged Pnc1. Actin was detected using an anti-actin antibody and used as a loading control. The relative ratio of Pnc1 to actin was normalized against that of WT cells and is shown below each lane. The positions of molecular-weight markers (in kDa) are indicated on the left of blots. Data are representative of at least three independent experiments.

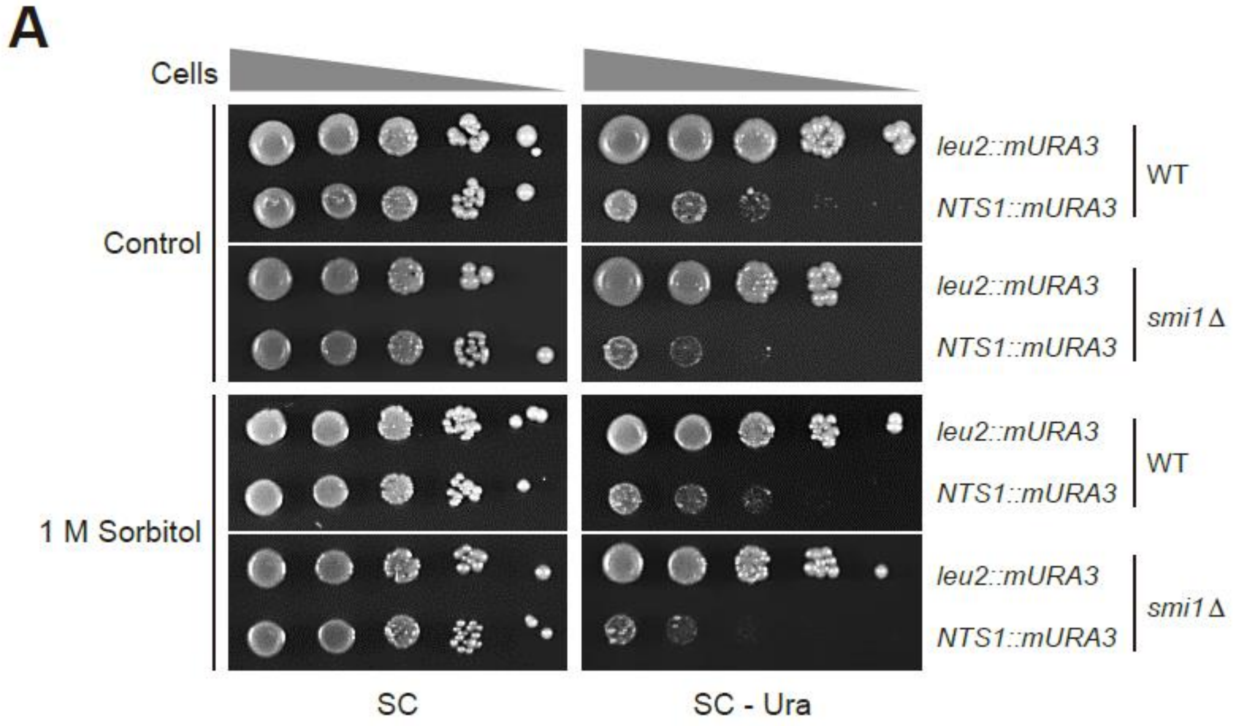

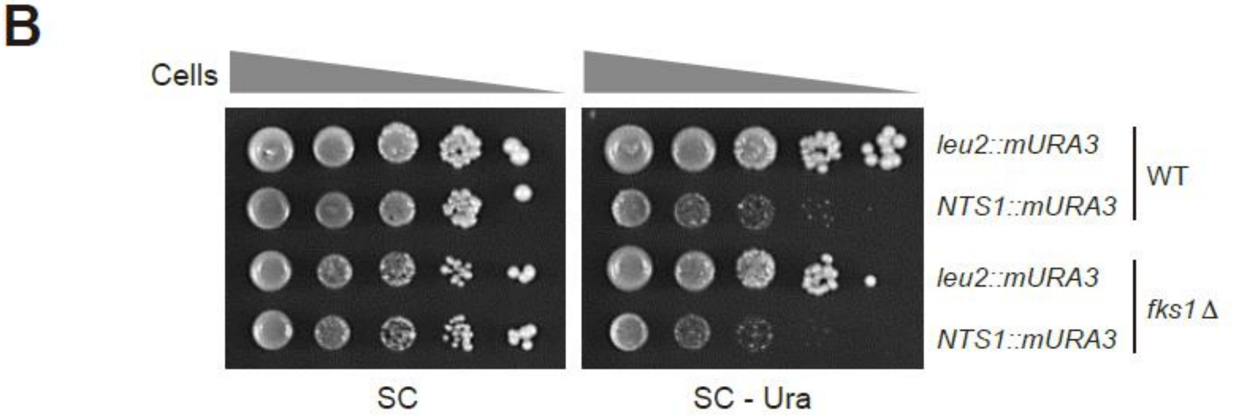

Figure S5. The role of Smi1 in rDNA silencing is not related to cell wall integrity. *A,* rDNA silencing assay was performed with wild-type (WT) and *smi1*Δ cells. Silencing at the rDNA region was assessed by monitoring the growth of tenfold serial dilution of cells on SC media lacking uracil in the presence or absence of 1 M sorbitol. SC medium was used as a plating control. *B,* rDNA silencing assay was performed with WT and *fks1*Δ cells. Silencing at the rDNA region was assessed by monitoring the growth of tenfold serial dilution of cells on SC media lacking uracil. SC medium was used as a plating control.

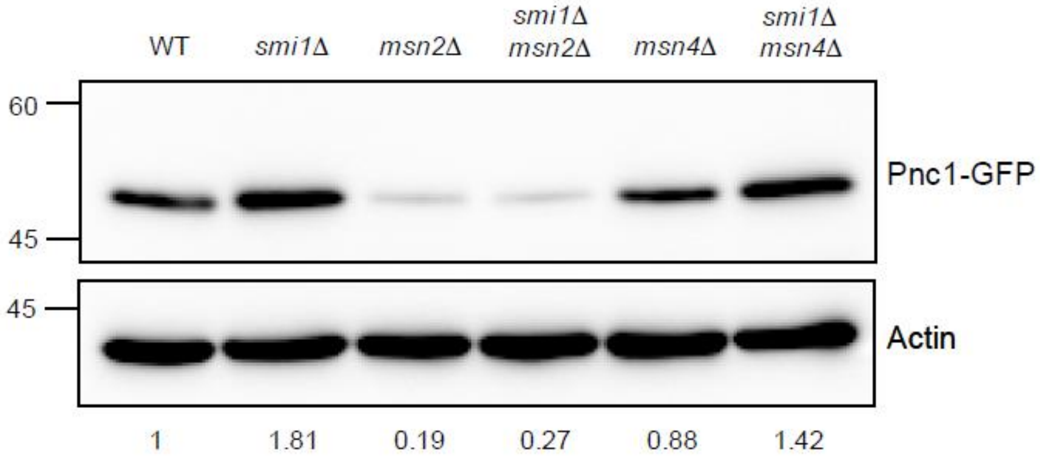

Figure S6. Msn4 plays a minor role in Pnc1 expression. Total protein was extracted from wild-type (WT), *smi1*Δ, *msn2*Δ, *smi1*Δ *msn2*Δ, *msn4*Δ, and *smi1*Δ *msn4*Δ cells, and immunoblotting was performed using a mouse anti-GFP antibody for the detection of GFP-tagged Pnc1 protein. Actin was detected using an anti-actin antibody and used as a loading control. The relative ratio of Pnc1 to actin was normalized against that of WT cells and is shown below each lane. The positions of molecular-weight markers (in kDa) are indicated on the left of blots. Data are representative of at least three independent experiments.

Table S1. Strains used in this study.

Strain	Genotype	Source
BY4741	*MAT*a *his3Δ1 leu2Δ0 met15Δ0 ura3Δ0*	EUROSCARF
HY2215	*MAT*a *his3Δ1 leu2Δ0 met15Δ0 ura3Δ0 smi1Δ::URA3*	This study
HY2254	*MAT*a *his3Δ1 leu2Δ0 met15Δ0 ura3Δ0 his3::SMI1-5xMYC*	This study
HY2255	*MAT*a *his3Δ1 leu2Δ0 met15Δ0 ura3Δ0 smi1Δ::URA3 his3::SMI1-5xMYC*	This study
HY2216	*MAT*a *his3Δ1 leu2Δ0 met15Δ0 ura3Δ0 sir2Δ::LEU2*	This study
HY2217	*MAT*a *his3Δ1 leu2Δ0 met15Δ0 ura3Δ0 smi1Δ::URA3 sir2Δ::LEU2*	This study
DMY2798	*MAT*a *ade2-1 ura3-1 trp1-1 leu2-3,112 his3-11 can1-100 leu2::mURA3*	(1)
DMY2804	*MAT*a *ade2-1 ura3-1 trp1-1 leu2-3,112 his3-11 can1-100 RDN1-NTS1::mURA3*	(1)
HY2218	*MAT*a *ade2-1 ura3-1 trp1-1 leu2-3,112 his3-11 can1-100 leu2::mURA3 smi1Δ::HIS3*	This study
HY2219	*MAT*a *ade2-1 ura3-1 trp1-1 leu2-3,112 his3-11 can1-100 RDN1-NTS1::mURA3 smi1Δ::HIS3*	This study
HY2256	*MAT*a *ade2-1 ura3-1 trp1-1 leu2-3,112 his3-11 can1-100 leu2::mURA3 his3::SMI1-5xMYC*	This study
HY2257	*MAT*a *ade2-1 ura3-1 trp1-1 leu2-3,112 his3-11 can1-100 RDN1-NTS1::mURA3 his3::SMI1-5xMYC*	This study
HY2258	*MAT*a *ade2-1 ura3-1 trp1-1 leu2-3,112 his3-11 can1-100 leu2::mURA3 smi1Δ::TRP1 his3::SMI1-5xMYC*	This study
HY2259	*MAT*a *ade2-1 ura3-1 trp1-1 leu2-3,112 his3-11 can1-100 RDN1-NTS1::mURA3 smi1Δ::TRP1 his3::SMI1-5xMYC*	This study
HY0245	*MAT*a *ade2-1 ura3-1 trp1-1 leu2-3,112 his3-11 can1-100 leu2::mURA3 sir2Δ::TRP1*	(2)
HY0291	*MAT*a *ade2-1 ura3-1 trp1-1 leu2-3,112 his3-11 can1-100 RDN1-NTS1::mURA3 sir2Δ::TRP1*	(2)
HY2220	*MAT*a *ade2-1 ura3-1 trp1-1 leu2-3,112 his3-11 can1-100 leu2::mURA3 smi1Δ::HIS3 sir2Δ::TRP1*	This study
HY2221	*MAT*a *ade2-1 ura3-1 trp1-1 leu2-3,112 his3-11 can1-100 RDN1-NTS1::mURA3 smi1Δ::HIS3 sir2Δ::TRP1*	This study
DMY3010	*MAT*a *ade2-1 ura3-1 trp1-1 leu2-3,112 his3-11 can1-100* $RAD5^{+}$ *with RDN1::ADE2*	(1)
HY2223	*MAT*a *ade2-1 ura3-1 trp1-1 leu2-3,112 his3-11 can1-100* $RAD5^{+}$ *with RDN1::ADE2 smi1Δ::URA3*	This study

HY2260	MATa ade2-1 ura3-1 trp1-1 leu2-3,112 his3-11 can1-100 $RAD5^+$ with RDN1::ADE2 his3::SMI1-5xMYC	This study
HY2261	MATa ade2-1 ura3-1 trp1-1 leu2-3,112 his3-11 can1-100 $RAD5^+$ with RDN1::ADE2 smi1Δ::URA3 his3::SMI1-5xMYC	This study
HY2222	MATa ade2-1 ura3-1 trp1-1 leu2-3,112 his3-11 can1-100 $RAD5^+$ with RDN1::ADE2 sir2Δ::TRP1	This study
HY2224	MATa ade2-1 ura3-1 trp1-1 leu2-3,112 his3-11 can1-100 $RAD5^+$ with RDN1::ADE2 smi1Δ::URA3 sir2Δ::TRP1	This study
HY1170	MATa his3Δ1 leu2Δ0 met15Δ0 ura3Δ0 MSN2-GFP-HIS3MX6	(3)
HY2225	MATa his3Δ1 leu2Δ0 met15Δ0 ura3Δ0 MSN2-GFP-HIS3MX6 smi1Δ::URA3	This study
HY1172	MATa his3Δ1 leu2Δ0 met15Δ0 ura3Δ0 MSN2-TAP-HIS3MX6	(4)
HY2226	MATa his3Δ1 leu2Δ0 met15Δ0 ura3Δ0 MSN2-TAP-HIS3MX6 smi1Δ::URA3	This study
HY1174	MATa his3Δ1 leu2Δ0 met15Δ0 ura3Δ0 PNC1-GFP-HIS3MX6	(3)
HY2227	MATa his3Δ1 leu2Δ0 met15Δ0 ura3Δ0 PNC1-GFP-HIS3MX6 smi1Δ::URA3	This study
HY1176	MATa his3Δ1 leu2Δ0 met15Δ0 ura3Δ0 PNC1-GFP-HIS3MX6 msn2Δ::KanMX4 msn4Δ::LEU2	(2)
HY2228	MATa his3Δ1 leu2Δ0 met15Δ0 ura3Δ0 PNC1-GFP-HIS3MX6 smi1Δ::URA3 msn2Δ::KanMX4 msn4Δ::LEU2	This study
HY1178	MATa his3Δ1 leu2Δ0 met15Δ0 ura3Δ0 SIR2-TAP-HIS3MX6	(4)
HY2233	MATa his3Δ1 leu2Δ0 met15Δ0 ura3Δ0 SIR2-TAP-HIS3MX6 smi1Δ::URA3	This study
HY1180	MATa his3Δ1 leu2Δ0 met15Δ0 ura3Δ0 SIR2-TAP-HIS3MX6 msn2Δ::KanMX4 msn4Δ::LEU2	(2)
HY2234	MATa his3Δ1 leu2Δ0 met15Δ0 ura3Δ0 SIR2-TAP-HIS3MX6 smi1Δ::URA3 msn2Δ::KanMX4 msn4Δ::LEU2	This study
HY1183	MATa his3Δ1 leu2Δ0 met15Δ0 ura3Δ0 msn2Δ::KanMX4 msn4Δ::LEU2	This study
HY2262	MATa his3Δ1 leu2Δ0 met15Δ0 ura3Δ0 smi1Δ::URA3 msn2Δ::KanMX4 msn4Δ::LEU2	This study
HY2263	MATa his3Δ1 leu2Δ0 met15Δ0 ura3Δ0 pnc1Δ::LEU2	This study
HY2264	MATa his3Δ1 leu2Δ0 met15Δ0 ura3Δ0 smi1Δ::URA3 pnc1Δ::LEU2	This study
HY1736	MATa his3Δ1 leu2Δ0 met15Δ0 ura3Δ0 pRS423-P_{CUP1}-6xMYC-$cki1^{2-200(S125/130A)}$	(5)
HY2235	MATa his3Δ1 leu2Δ0 met15Δ0 ura3Δ0 smi1Δ::LEU2 pRS423- P_{CUP1}-6xMYC-$cki1^{2-200(S125/130A)}$	This study

HY1730	*MAT*a *his3Δ1 leu2Δ0 met15Δ0 ura3Δ0 pRS416; SCH9^{T570A}-5HA*	(5)
HY2236	*MAT*a *his3Δ1 leu2Δ0 met15Δ0 ura3Δ0 smi1Δ::LEU2 pRS416; SCH9^{T570A}-5HA*	This study
HY2209	*MAT*a *his3Δ1 leu2Δ0 met15Δ0 ura3Δ0 HOG1-GFP-HIS3MX6*	(3)
HY2237	*MAT*a *his3Δ1 leu2Δ0 met15Δ0 ura3Δ0 HOG1-GFP-HIS3MX6 smi1Δ::LEU2*	This study
HY2238	*MAT*a *his3Δ1 leu2Δ0 met15Δ0 ura3Δ0 MSN2-GFP-HIS3MX6 hog1Δ::LEU2*	This study
HY2239	*MAT*a *his3Δ1 leu2Δ0 met15Δ0 ura3Δ0 MSN2-GFP-HIS3MX6 smi1Δ::URA3 hog1Δ::LEU2*	This study
HY2265	*MAT*a *his3Δ1 leu2Δ0 met15Δ0 ura3Δ0 MSN2-GFP-HIS3MX6 pRS416*	This study
HY2266	*MAT*a *his3Δ1 leu2Δ0 met15Δ0 ura3Δ0 MSN2-GFP-HIS3MX6 smi1Δ::LEU2 pRS416*	This study
HY2267	*MAT*a *his3Δ1 leu2Δ0 met15Δ0 ura3Δ0 MSN2-GFP-HIS3MX6 hog1Δ::KanMX4 pRS416*	This study
HY2268	*MAT*a *his3Δ1 leu2Δ0 met15Δ0 ura3Δ0 MSN2-GFP-HIS3MX6 smi1Δ::LEU2 hog1Δ::KanMX4 pRS416*	This study
HY2269	*MAT*a *his3Δ1 leu2Δ0 met15Δ0 ura3Δ0 MSN2-GFP-HIS3MX6 pRS416-P_{ADH1}-HOG1-5xMYC*	This study
HY2270	*MAT*a *his3Δ1 leu2Δ0 met15Δ0 ura3Δ0 MSN2-GFP-HIS3MX6 smi1Δ::LEU2 pRS416-P_{ADH1}-HOG1-5xMYC*	This study
HY2271	*MAT*a *his3Δ1 leu2Δ0 met15Δ0 ura3Δ0 MSN2-GFP-HIS3MX6 hog1Δ::KanMX4 pRS416-P_{ADH1}-HOG1-5xMYC*	This study
HY2272	*MAT*a *his3Δ1 leu2Δ0 met15Δ0 ura3Δ0 MSN2-GFP-HIS3MX6 smi1Δ::LEU2 hog1Δ::KanMX4 pRS416-P_{ADH1}-HOG1-5xMYC*	This study
HY2240	*MAT*a *his3Δ1 leu2Δ0 met15Δ0 ura3Δ0 PNC1-GFP-HIS3MX6 hog1Δ::LEU2*	This study
HY2241	*MAT*a *his3Δ1 leu2Δ0 met15Δ0 ura3Δ0 PNC1-GFP-HIS3MX6 smi1Δ::URA3 hog1Δ::LEU2*	This study
HY2273	*MAT*a *his3Δ1 leu2Δ0 met15Δ0 ura3Δ0 PNC1-GFP-HIS3MX6 pRS416*	This study
HY2274	*MAT*a *his3Δ1 leu2Δ0 met15Δ0 ura3Δ0 PNC1-GFP-HIS3MX6 smi1Δ::LEU2 pRS416*	This study
HY2275	*MAT*a *his3Δ1 leu2Δ0 met15Δ0 ura3Δ0 PNC1-GFP-HIS3MX6 hog1Δ::KanMX4 pRS416*	This study
HY2276	*MAT*a *his3Δ1 leu2Δ0 met15Δ0 ura3Δ0 PNC1-GFP-HIS3MX6 smi1Δ::LEU2 hog1Δ::KanMX4 pRS416*	This study
HY2277	*MAT*a *his3Δ1 leu2Δ0 met15Δ0 ura3Δ0 PNC1-GFP-HIS3MX6 pRS416-P_{ADH1}-HOG1-5xMYC*	This study
HY2278	*MAT*a *his3Δ1 leu2Δ0 met15Δ0 ura3Δ0 PNC1-GFP-HIS3MX6 smi1Δ::LEU2 pRS416-P_{ADH1}-HOG1-5xMYC*	This study

HY2279	*MAT*a *his3Δ1 leu2Δ0 met15Δ0 ura3Δ0 PNC1-GFP-HIS3MX6 hog1Δ::KanMX4 pRS416-*P_{ADH1}*-HOG1-5xMYC*	This study
HY2280	*MAT*a *his3Δ1 leu2Δ0 met15Δ0 ura3Δ0 PNC1-GFP-HIS3MX6 smi1Δ::LEU2 hog1Δ::KanMX4 pRS416-*P_{ADH1}*-HOG1-5xMYC*	This study
HY2281	*MAT*a *his3Δ1 leu2Δ0 met15Δ0 ura3Δ0 hog1Δ::LEU2*	This study
HY2282	*MAT*a *his3Δ1 leu2Δ0 met15Δ0 ura3Δ0 smi1Δ::URA3 hog1Δ::LEU2*	This study
HY2244	*MAT*a *ade2-1 ura3-1 trp1-1 leu2-3,112 his3-11 can1-100 leu2::mURA3 fks1Δ::HIS3*	This study
HY2245	*MAT*a *ade2-1 ura3-1 trp1-1 leu2-3,112 his3-11 can1-100 RDN1-NTS1::mURA3 fks1Δ::HIS3*	This study
HY2229	*MAT*a *his3Δ1 leu2Δ0 met15Δ0 ura3Δ0 PNC1-GFP-HIS3MX6 msn2Δ::LEU2*	This study
HY2230	*MAT*a *his3Δ1 leu2Δ0 met15Δ0 ura3Δ0 PNC1-GFP-HIS3MX6 smi1Δ::URA3 msn2Δ::LEU2*	This study
HY2231	*MAT*a *his3Δ1 leu2Δ0 met15Δ0 ura3Δ0 PNC1-GFP-HIS3MX6 msn4Δ::LEU2*	This study
HY2232	*MAT*a *his3Δ1 leu2Δ0 met15Δ0 ura3Δ0 PNC1-GFP-HIS3MX6 smi1Δ::URA3 msn4Δ::LEU2*	This study

Table S2. Oligonucleotide primers used for quantitative real-time PCR in this study.

Gene	Forward Primer	Reverse Primer
mURA3	CTGTTGACATTGCGAAGAGC	TCTCCCTTGTCATCTAAACC
PNC1	AGACGAGGTTTACATTGTCG	CCTTCAACTCTTCCTTAACC
ACT1	TGACTGACTACTTGATGAAG	TGCATTTCTTGTTCGAAGTC

Table S3. Oligonucleotide primers used for ChIP assays in this study.

Region	Forward Primer	Reverse Primer
PNC1 promoter	GATCAAGGTGGCACACAGGG	ATACATAGTGGGCCAAACGG
rDNA-25S	CGACTAACCCACGTCCAACT	CCGAATGAACTAGCCCTGAA
rDNA-NTS1	TCCCCACTGTTCACTGTTCA	AGGGCTTTCACAAAGCTTCC
rDNA-NTS2/18S	AAGATGCCCACGATGAGACT	GGGAGGTACTTCATGCGAAA
rDNA-18S	CCAGAACGTCTAAGGGCATC	CTCACCAGGTCCAGACACAA

References

1. Huang, J., Brito, I. L., Villen, J., Gygi, S. P., Amon, A., and Moazed, D. (2006) Inhibition of homologous recombination by a cohesin-associated clamp complex recruited to the rDNA recombination enhancer. *Genes Dev* **20**, 2887-2901
2. Ha, C. W., Kim, K., Chang, Y. J., Kim, B., and Huh, W. K. (2014) The beta-1,3-glucanosyltransferase Gas1 regulates Sir2-mediated rDNA stability in Saccharomyces cerevisiae. *Nucleic Acids Res* **42**, 8486-8499
3. Huh, W.-K., Falvo, J. V., Gerke, L. C., Carroll, A. S., Howson, R. W., Weissman, J. S., and O'shea, E. K. (2003) Global analysis of protein localization in budding yeast. *Nature* **425**, 686-691
4. Ghaemmaghami, S., Huh, W.-K., Bower, K., Howson, R. W., Belle, A., Dephoure, N., O'shea, E. K., and Weissman, J. S. (2003) Global analysis of protein expression in yeast. *Nature* **425**, 737-741
5. Yi, D. G., Hong, S., and Huh, W. K. (2018) Mitochondrial dysfunction reduces yeast replicative lifespan by elevating RAS-dependent ROS production by the ER-localized NADPH oxidase Yno1. *PLoS One* **13**, e0198619
